# Role of Coagulation Factors in Hepatocellular Carcinoma: A Literature Review

**DOI:** 10.3390/life15010034

**Published:** 2024-12-30

**Authors:** Azeem Azam, Aleksandra Klisic, Filiz Mercantepe, Hamza Faseeh, Tolga Mercantepe, Saira Rafaqat

**Affiliations:** 1 Institute of Zoology, University of the Punjab, Lahore 54590, Pakistan; azeemazam360@gmail.com; 2 Faculty of Medicine, University of Montenegro, 81000 Podgorica, Montenegro; 3 Center for Laboratory Diagnostics, Primary Health Care Center, 81000 Podgorica, Montenegro; 4 Department of Endocrinology and Metabolism, Faculty of Medicine, Recep Tayyip Erdogan University, Rize 53200, Türkiye; filiz.mercantepe@saglik.gov.tr; 5 Department of Zoology, Govt. Islamia Graduate College Civil Lines, Lahore 54000, Pakistan; kaifury12011@gmail.com; 6 Department of Histology, Faculty of Medicine, Recep Tayyip Erdogan University, Rize 53200, Türkiye; 7 Department of Zoology, Lahore College for Women University, Lahore 44444, Pakistan; saera.rafaqat@gmail.com

**Keywords:** hepatocellular carcinoma, coagulation factors, pathophysiology

## Abstract

Hepatocyte carcinoma (HCC) is a globally prevalent neoplasm with profound effects on morbidity and mortality rates. This review summarizes the complex interactions between coagulation abnormalities and the pathophysiological mechanisms underlying HCC. Essential coagulation biomarkers, such as P-selectin, thrombomodulin, d-dimer, prothrombin, and von Willebrand factor, are reviewed for their diagnostic, prognostic, and therapeutic significance. The contribution of these biomarkers to tumor progression, metastatic spread, and patient prognosis is highlighted through a synthesis of contemporary research findings. In addition, this review highlights the underlying mechanisms linking coagulation pathways to HCC pathogenesis and explores potential therapeutic targets. An integrative perspective on the role of coagulation markers in HCC may improve clinical management strategies for patients affected by this malignancy.

## 1. Introduction

Hepatocellular carcinoma (HCC) is currently the fifth most common cancer worldwide and a serious public health concern due to its increasing incidence. Even though HCC is the most frequent cause of primary liver cancer, secondary liver cancers, or metastases from other tumors, are far more prevalent and account for around 90% of all liver cancer cases [1]. The most common tumors that metastasize to the liver are those of the digestive system, especially colorectal malignancies [2]. In the oncological care of cancer patients, liver metastasis is a significant therapeutic target since it limits long-term survival for a considerable number of patients [3,4].

The hemostatic system is impacted by malignancy, and malignancy is impacted by the hemostatic system. There are several coagulation disorders in cancer patients, which serve as the foundation for their higher risk of bleeding and thrombosis. The reasons for this coagulation impairment depend on both cancer-specific and general risk factors that are shared by other patient groups. There are several connections between the hemostatic elements and the biology of cancer. Hemostatic factors contribute to the growth of tumors even though cancer cells can activate the coagulation system [5].

Liver function is directly related to hemostasis since the reticuloendothelial system of the liver is essential for eliminating activation products. The majority of coagulation factors are produced by histological cells in the liver. Coagulation issues may vary in severity according to the degree of liver function impairment. Prothrombin; factors VII, IX, and X; proteins C and S are examples of vitamin K-dependent factors that might show declines in acute or chronic hepatocellular disorders, while other parameters stay intact. In addition to disseminated intravascular coagulation, patients with hepatic failure may exhibit a whole range of factor deficits [6].

Chronic liver disease and HCC can interfere with the coagulation system. In individuals with HCC, thrombophilic abnormalities are common, and their risk of venous thromboembolism is significantly elevated [7]. Reduced blood levels of clotting factors II, V, VII, IX, X, and antithrombin III in individuals with liver disease are mostly caused by the liver’s diminished ability to produce proteins. Consequently, as long as other factors that might affect the blood level are properly taken into account, measuring the activity or concentration of these coagulation proteins is a valuable test of liver function and a prognostic indicator. Assays for clotting factors are only marginally useful for a liver disease differential diagnosis [8].

Clinical investigation has demonstrated a strong correlation between alterations in coagulation and proteolysis variables and the development and progression of cancer. In cancer tissues and plasma from patients with HCC, the expression of tissue factor (TF), urokinase-type plasminogen activator (uPA), and urokinase-type plasminogen activator receptor (uPAR) were found, and their predictive value was examined. In HCC invasion and metastasis, TF, uPA, and uPAR may work in concert and may be related to prognosis [9].

The present review article summarizes the role of coagulation factors in the pathophysiology of HCC, including P-selectin, thrombomodulin, d-dimer, prothrombin, von Willebrand factor, fibrinogen and fibrin, tissue factor, tissue plasminogen activator, plasminogen activator inhibitor-1, soluble urokinase-type plasminogen activator receptor, and antithrombin, as explained in figures and tables. Several databases, including Science Direct, PubMed, and Google Scholar, were used to conduct the literature review. Hepatocellular carcinoma, coagulation factors, and pathophysiology were among the precise terms included in the search, which ran until 20 October 2024. Clinical research was restricted to English-language publications. No time limit was set, even though current research was highlighted.

## 2. Pathophysiological Aspects of Coagulation Factors in HCC

### 2.1. P-Selectin

The platelet alpha granule and the Weibel–Palade body of endothelial cells contain the adhesion protein P-selectin, which helps blood cells roll on the endothelium surface. At locations of inflammation and tissue damage, it starts circulating leukocytes to adhere to platelets, endothelial cells, and other leukocytes. P-selectin ligands on the surface of platelets and endothelial cells enable this process by sulfating a particular tyrosine residue at the N-terminal for P-selectin recognition. One of the main ligands for P-selectin that causes leukocytes to roll on active endothelium is P-selectin glycoprotein ligand-1 (PSGL-1). Platelet adhesion and aggregation are facilitated by sulfatides, which bind to P-selectin. In healthy people, soluble P-selectin (sP-selectin) is found in the blood at concentrations of around 100 ng/mL. Increased P-selectin expression and elevated sP-selectin levels are possible indicators for several cancers, such as blood cancer, and breast, renal, colon, and neoplastic pulmonary illnesses [10].

HCC patients frequently have venous thromboembolism (VTE). Both malignant and non-malignant causes of chronic liver disease are linked to its pathomechanism. There are probably other reasons associated with this high occurrence. Individuals with VTE and HCC have a reduced survival duration compared to those without VTE. The Von Willebrand factor may be bound with a high affinity by factor VIII, an essential procoagulant protein. In individuals with normal factor VIII, the risk of venous thrombosis may be roughly six times higher if the rise in factor VIII is more than 150%. Pregnancy, inflammation, and advancing age all cause an increase in its level [11].

Patients with normal levels (4%), as opposed to those with increased FVIII activity (14%), had a significantly higher proportion of VTE. Membrane glycoproteins known as selectins are implicated in cell adhesion. P-selectin is thought to increase the risk of VTE in cancer patients. VTE risk may be identified in patients based on the amount of soluble P-selectin at the time of cancer diagnosis. Factor VIII, P-selectin in plasma, and CD62 expression measured by flow cytometry are the most reliable indicators of the risk of VTE in patients with HCC. These findings lead us to regard FVIII and P-selectin as prognostic biomarkers for VTE [11].

In patients with HCC, the hypercoagulable state driven by liver dysfunction, systemic inflammation, and tumor-related factors increases the risk of not only VTE but also arterial thrombotic complications such as ischemic stroke and acute coronary syndrome (ACS). There is ongoing discussion over the relationship between liver cirrhosis and coronary artery disease. In individuals with liver cirrhosis, atherosclerotic lesions have been linked to common pathogenic pathways, such as vascular inflammation, endothelial dysfunction, and a procoagulant state [12].

Systemic inflammation and hypercoagulability can trigger coronary artery thrombosis. Endothelial dysfunction and increased platelet activation promote atherosclerosis and plaque rupture, leading to ACS (e.g., myocardial infarction). Hypofibrinolysis, seen in chronic liver disease, prolongs clot stability in coronary arteries. These complications significantly impact prognosis and require careful management to balance the risks of thrombosis and bleeding. Recognizing these events early and addressing cardiovascular risk factors are critical to improving patient outcomes [12].

### 2.2. Thrombomodulin

Important for many biological functions, thrombomodulin (TM) is a type-I transmembrane protein that is mostly expressed in endothelial cells. Biofluids such as blood and urine include many types of circulating TM. Comprising different TM domains, soluble TM (sTM) is the most common circulating type. It is created under a variety of circumstances by cleaving the intact protein chemically or enzymatically. Under healthy conditions, soluble TM is present in the blood at low levels. However, several clinical conditions, such as cardiovascular, inflammatory, infection, and metabolic illnesses, that are associated with endothelial dysfunction cause its levels to rise [13].

TM is an essential molecule that mediates the homeostasis of circulation by interacting with thrombin. A tight clot can be formed by the TM-thrombin complex activating protein C and thrombin-activatable fibrinolysis inhibitor. The reduced TM expression in several cancer tissues may be associated with the spread of the malignancy. It is currently unknown, therefore, how TM contributes to the development of HCC. It used Western blotting and a real-time polymerase chain reaction to assess the expression of TM in HCC cells (HepJ5 and skHep-1 cells). Next, it used TM-specific short hairpin RNA (shRNA) that is overexpressed in HCC cells to modify the expression of TM. To track HCC cells’ capacity to migrate at various levels of TM expression, the transwell migration test was used [14].

Both at the transcriptional and translational levels, it was discovered that TM was ectopically strongly expressed in skHep-1. It observed a significant increase in the capacity for metastasis in skHep-1 cells following the suppression of TM expression. On the other hand, HepJ5 cells’ capacity to spread was reduced when TM was overexpressed. It revealed that the overexpression of zinc finger E-box binding homeobox-1 (ZEB1), an E-cadherin regulator, was necessary for the reduced TM-mediated improvement of cell migration. In HCC, TM could regulate the spread of malignancy. ZEB1 may rise and E-cadherin levels might fall as a result of downregulating the TM expression [14].

To activate protein C, TM changes thrombin from a procoagulant to an anticoagulant protein. Thrombin is also crucial for the cancer cells’ ability to spread throughout the body. In 141 instances of resected HCC with a diameter of less than 6 cm, it conducted an immunohistochemical and clinicopathological analysis of TM. A total of 25 specimens (17.73%) had positive TM staining. Cancer cell surfaces and cytoplasm were both shown to have TM. Examined in HCC are the clinicopathological results based on the positive TM test. Patients whose tissue stained positive for TM had preoperative plasma TM levels that were considerably greater than those of patients whose tissue stained negative; there were no differences between the two groups’ postoperative TM levels [15].

Furthermore, compared to individuals whose tissue tested negative for TM, the incidences of intrahepatic metastases, tumor thrombus in the portal vein, and capsular infiltration were much lower in the TM-positive patient group. Compared to patients whose tissue tested negatively for TM, those whose tissue stained positively for TM had a considerably greater recurrence freedom rate. Thus, the intrahepatic dissemination of TM-producing HCC is inactive. Consequently, TM may, by its anticoagulant action, limit tumor cell adherence to the portal vein and thereby stop intrahepatic metastases from spreading [15].

### 2.3. d-Dimer

d-dimer is a soluble by-product of fibrin degradation, which arises from the fibrinolytic system’s systematic disintegration of thrombi. Several research works have emphasized the importance of d-dimer as a useful marker of coagulation and fibrinolysis activation. Consequently, d-dimer has been thoroughly investigated and is frequently used for the diagnosis of venous thromboembolism. In addition, d-dimer has been evaluated to determine the ideal duration of anticoagulation in patients with venous thromboembolism, diagnose and track disseminated intravascular coagulation, and aid in the identification of medical patients at a heightened risk for venous thromboembolism. Consequently, the measurement of d-dimer levels is essential for directing therapeutic decisions [16].

It is unknown how useful d-dimer is in determining the therapeutic effectiveness of drug-eluting beads transarterial chemoembolization (DEB-TACE), although it does show some predictive value in patients with HCC who had hepatectomy and microwave ablation. Therefore, another study looks at how d-dimer and tumor characteristics, response, and survival after DEB-TACE in patients with HCC are correlated. In HCC patients, elevated d-dimer levels were associated with larger tumor sizes, more tumor nodules, higher Child–Pugh stages, and portal vein invasion. Furthermore, d-dimer levels increased while receiving DEB-TACE treatment. While further extensive research validation is necessary, d-dimer may be useful for tracking the prognosis of DEB-TACE treatment in HCC [17].

Severe consequences from HCC include portal vein thrombosis (PVT). Hepatic cirrhosis and HCC patients have poorer prognoses because of PVT’s deteriorating effects on the liver and the increased risk of bleeding from its malfunction. Whether d-dimer testing may be a sensitive sign for the diagnosis and prognosis of PVT patients with HCC was the purpose of the investigation. Between HCC patients with PVT and those without PVT, *p* < 0.002 and *p* < 0.001, respectively, d-dimer levels were considerably higher. The detection of active coagulation, which might show up in HCC with PVT is made possible by the sensitive fibrin turnover marker plasma d-dimer [18].

High sensitivity and specificity plasma d-dimer was anticipated to be a significant plasma marker for the clinical diagnosis of HCC [19].

Because of their elevated plasma d-dimer, hypercoagulatory and hyperfibrinolytic states, and other factors, HCC patients frequently have aberrant blood coagulation function. d-dimer levels may fluctuate in HCC patients receiving locoregional therapy. In HCC, a progressive increase in d-dimer levels four months after locoregional therapy was linked to tumor growth, indicating aggressive tumor biology and a poor prognosis. Elevated d-dimer levels before and after locoregional treatment may be indicators of a disapproving tumor profile and a poor prognosis. Variations in plasma d-dimer levels can be utilized as a potential prognostic biomarker in predicting the efficacy of locoregional treatment in HCC patients [20].

The third most prevalent cause of cancer-related mortality globally and the fifth most common kind of cancer is HCC. Patients with asymptomatic HCC are discovered at an advanced stage, which results in a dismal prognosis because there are ineffective instruments for early identification. These days, AFP testing is not useful for screening HCC in patients with AFP-negative hepatocellular carcinoma (AFP-NHCC), which has been detected in a large number of HCC patients. Another reported the clinical relevance of pre-albumin (PA), fibrinogen, and d-dimer expression patterns in AFP-NHCC. The plasma d-dimer levels in the controls were considerably lower than those of AFP-NHCC. PA, fibrinogen, and d-dimer expression levels were important in the carcinogenesis of AFP-NHCC. Furthermore, d-dimer and PA may be seen as possible diagnostic markers in AFP-NHCC [21].

There is evidence linking the activation of fibrinolysis and coagulation to angiogenesis, tumor cell invasion, tumor growth, and metastasis. A biomarker that universally signifies the initiation of hemostasis and fibrinolysis is d-dimer. The literature studies investigate how the tumor grade in HCV-related HCC is correlated with the amount of circulating d-dimer. Following the completion of a metastatic work-up, the HCC was graded as follows: grade one for nodules smaller than 5 cm or up to three nodules, with the largest being less than 3 cm and not involving vascular invasion or extra-hepatic involvement; grade two for nodules larger than 5 cm or beyond the Milan criteria, and grade three for nodules that were metastatic (vascular invasion, lymph node metastasis, and distant metastasis) [22].

Significant positive correlations were seen between the circulating d-dimer level and the size and grade of HCC. Additionally, the amount of circulating d-dimer in HCV-related HCC was found to be strongly linked with both the grade of the HCC and the highest tumor size for both BCLC stage D and HCC grades 2 and 3. An HCC grade above the Milan criteria can be predicted by a cutoff level of a circulating d-dimer level of ≥300 ng/mL [22].

### 2.4. Prothrombin

The human prothrombin-encoding F2 gene is located at location 11.2 on the short arm of chromosome 11. With a single pre/pro-polypeptide of 622 amino acids, hepatocytes in the liver produce prothrombin, a crucial protein in blood-clotting processes. Its significance in normal physiological functions cannot be overstated. In terms of health, diminished activity is linked to hemorrhagic diseases, while excessive activity is associated with thrombosis. Therefore, understanding this molecule is crucial. Prothrombin, isolated from plasma, serves as the precursor for two enzymes: thrombin, responsible for clotting fibrinogen, and auto prothrombin C, which facilitates prothrombin activation. Besides these enzymes, at least three prothrombin activation intermediates exist, namely, auto prothrombin I, auto prothrombin II, and prothrombin-R [23].

Prothrombin induced by vitamin K absence-II (PIVKA-II), also known as des-gamma-carboxy prothrombin or des-γ-carboxyl prothrombin (DCP), is an abnormal protein produced in hepatocellular carcinoma. The absence of glutamic acid (Glu) residues made the coagulation function inadequate. PIVKA-II elevation was linked to malignant behavior in terms of invasion, metastasis, and proliferation. To elucidate the process, three main signaling routes were postulated. Four areas of HCC management might be improved by PIVKA-II: cost-effectiveness, low invasiveness, ease of use, and effectiveness [24].

To enhance HCC surveillance in high-risk individuals, PIVKA-II proved to be a proficient and adaptable instrument. Before the advent of imaging, variations in PIVKA-II serum levels offered important molecular modification information. The use of PIVKA-II in conjunction with other biomarkers performed better than conventional diagnostic techniques. Thirdly, in HCC, PIVKA-II served as an index for evaluating the therapy response. Measurements were taken after surgery to determine the effectiveness of the therapy and to help with preoperative therapy selection. Fourth, PIVKA-II was regarded as a potential indicator of HCC prognosis. Microvascular invasion, metastasis, and recurrence were related to a higher risk of PIVKA-II in these patients [24].

As a paracrine factor that integrates HCC with vascular endothelial cells and an autologous growth factor that promotes HCC development, DCP’s biologically malignant potential has recently been clarified. It has been discovered that DCP promotes HCC development by activating the DCP–Met–JAK1–STAT3 signaling pathway. By activating matrix metalloproteinase (MMP) and the ERK1/2 MAPK signaling pathway, DCP may promote HCC invasion and metastasis. Furthermore, DCP is essential for the development of angiogenesis. DCP may enhance the angiogenic factors secreted by vascular endothelial cells and HCC. The DCP–KDR–PLC–γ–MAPK signaling pathway may be activated in response to DCP’s effects on angiogenesis [25].

Recently, there has been a lot of interest in using DCP as a predictor of the risk of HCC recurrence after liver transplantation (LT), especially since the eligibility criteria for LT were recently expanded to include these patients. The results showed a high rate of homogeneity, confirming that the tumor marker DCP is a useful predictive factor, indicating a five-fold increased risk for HCC recurrence after liver transplant. With the assistance of DCP, LT eligibility criteria for patients with HCC are being enhanced. Based only on Japanese studies in the setting of living-donor LT, this knowledge needs further validation in the Western world [26].

Another study clarifies the clinical significance of des-γ-carboxy prothrombin levels in both liver tissues and HCC, with a special focus on the association between DCP levels in non-cancerous liver parts and the multicentric occurrence of HCC. The DCP level in the liver tissue was significantly higher in people with a multicentric incidence of HCC than in people without this symptom. The DCP level in HCC and the logarithm of the plasma DCP level are associated [27]. The DCP level in HCC did not significantly correlate with other clinicopathological parameters. When multicentric HCC occurred concurrently with non-cancerous liver sections, the DCP level in those liver sections was substantially greater than in the liver without multicentric HCC. Moreover, among the several clinicopathological variables, the DCP level in non-cancerous liver regions was one of the most significant and predicted determinants of the multicentric development of HCCs. Accordingly, the DCP level could be crucial in the development of hepatocarcinogenesis [27].

One of the primary causes of LT is hepatocellular carcinoma. Alpha-fetoprotein (AFP) and des-gamma-carboxy-prothrombin are two biomarkers that might be useful in determining the probability of recurrence after liver transplantation. In patients receiving LT for HCC, the purpose of another study is to assess the relationship between serum DCP levels and the degree of DCP immunohistochemistry labelling [28].

It happened during the first three months following transplantation in the negative group, but only in the positive groups did late recurrence occur. A more aggressive tumor profile and serum levels are correlated with DCP labelling in liver lesions. To what extent heavily DCP-labelled tumors enable the more accurate screening of high-risk patients before LT requires more research [28].

A well-known tumor marker for HCC is des-γ-carboxyl prothrombin. It was shown that DCP causes HCC cell lines to proliferate. In a dose-dependent manner, purified DCP induced the production of DNA in Hep3B and SK-Hep-1 cells. It has been shown that DCP binds to the cell surface receptor Met, triggering Met autophosphorylation. Additionally, DCP activates the STAT3 signaling pathway using Janus kinase 1 [29].

An analysis using luciferase gene reporter demonstrated that DCP stimulated transcription linked to STAT3. DCP-induced cell proliferation was inhibited by small interfering RNAs directed against both STAT3 and Met. The phosphoinositide 3-kinase/Akt pathway, Myc signaling pathway, and mitogen-activated protein kinase pathway were all unaffected by DCP. Based on these findings, it hypothesizes that DCP functions for HCC cell lines as an autologous mitogen. A significant signaling mechanism for DCP-induced cell proliferation might be the Met–Janus kinase 1–STAT3 pathway [29].

In patients with chronic hepatitis B virus (HBV) infection, the significance of blood prothrombin caused by the vitamin K absence or antagonist-II in the early diagnosis of HCC remains unclear. The study’s objectives were to assess PIVKA-II’s sensitivity and specificity for the identification of HCC during surveillance, either by itself or in conjunction with AFP, and to ascertain whether PIVKA-II posed a meaningful risk factor for patient survival. PIVKA-II’s sensitivity rate was 51.9% while AFP’s sensitivity rate was 57.5%. Following multivariate analysis, PIVKA-II was not a statistically significant risk factor for patient survival. In individuals with persistent HBV infection, PIVKA-II can be utilized as a tumor marker to identify HCC early on, particularly when combined with AFP [30].

### 2.5. Von Willebrand Factor

The main source of the large glycoprotein von Willebrand factor (vWF) is endothelial cells. vWF is essential for promoting platelet adhesion and aggregation at sites of vascular injury in addition to its function as a coagulation factor VIII carrier. These functions depend on the structure of vWF, which is made up of low-, intermediate-, and high-molecular-weight (LMW, IMW, and HMW) multimers of different sizes [31].

For individuals with chronic hepatitis and liver cirrhosis, several non-invasive biomarkers are available to diagnose the stage of liver fibrosis and predict the development of HCC. These biomarkers, however, lack enough accuracy. VWF has been associated with both apoptosis and angiogenesis in recent times. Moreover, VWF and HCC are linked to a hepatic spare capacity [32].

Another study investigated whether VWF may be a biomarker for the development of HCC and liver fibrosis. Those who had developed HCC or were in a severe state of liver fibrosis had greater VWF antigen (VWF: Ag) levels than those who had not. At a stage of severe liver fibrosis, the VWF: Ag area under the curve was 0.721. There was just one prognostic biomarker for the development of HCC, which, according to the multivariable analysis, was VWF: Ag. VWF: Ag was associated with liver fibrosis; it might help anticipate the emergence of HCC. To identify severe liver fibrosis and forecast the emergence of HCC, VWF is a potentially helpful biomarker [32].

The glycoprotein known as VWF is produced and released by megakaryocytes and vascular endothelial cells. It is present on the plasma membrane, endothelial cells, and platelet α-granules. The prevention or protesting of HCC in patients with chronic hepatitis B (CHB) may also be mediated by VWF, according to mounting data from many therapeutic trials. Even though it is yet unknown how VWF protects from or protests against HCC, more research is needed. VWF’s involvement in the growth of HCC, its functional domain in cancer, and VWF’s interactions with platelets and miRNAs are also investigated. The obstacles and future developments of VWF research are also anticipated in this paper [33].

HCC is the sixth most prevalent malignancy globally and is significantly predisposed by persistent hepatitis B virus (HBV) infection. Proteins in plasma from patients with HBV-associated HCC, nonmalignant cirrhosis, and chronic hepatitis B, and healthy persons were analyzed using iTRAQ in conjunction with mass spectrometry to investigate possible biomarkers for HCC. When comparing HCC patients to non-tumor controls, 21 aberrantly expressed proteins were found [34].

Moreover, the interferon signaling pathway was used to suppress HBV replication by a factor of more than two by siRNA-induced vWF silencing, which also hindered HCC cell invasion and migration in vitro. According to these findings, vWF may be used as a biomarker and maybe as a different target for therapeutic intervention to stop the progression of HCC and HBV infection [34].

A non-invasive predictor of portal hypertension, vWF-Ag is a poor prognostic indicator for several cancers. After a hepatectomy, increased portal hypertension is linked to worse postoperative morbidity and shorter survival times. This investigation sought to ascertain the relationship between vWF-Ag, surgical morbidity, and oncological prognosis. The resected tissues showed a strong connection between the vWF-Ag levels and the size of the tumor [35].

Individuals with preoperative vWF-Ag levels were considerably higher than those without any grade of postoperative problem. In patients with high vWF-Ag levels, the median overall survival was 39.8 months; in those with low levels, it was 73.4 months. Notably, there was a notable difference in the proportion of patients with low vWF-Ag levels versus high vWF-Ag levels who died of HCC. Given its strong correlation with tumor size, the risk of surgical complications, and long-term prognosis, vWF-Ag might be used as a prognostic marker for patients having a liver resection for HCC [35].

Patients with HCC following liver resection have three outcomes to consider: the recurrence of the illness, complications from portal hypertension, and posthepatectomy liver failure (PHLF). Another study assessed the von Willebrand factor antigen as a predictive biomarker for overall survival (OS) and time to recurrence (TTR) in clinically significant portal hypertension (CSPH) in a non-invasive manner [36].

The results of receiver operating characteristic (ROC) studies showed that vWF-Ag and indocyanine green clearance were equally predictive of PHLF, whereas the prediction of future liver remnants was less accurate (AUC, 0.756). vWF-Ag was associated with TTR and OS according to the Cox regression analysis. It confirmed the predictive efficacy of vWF-Ag for OS and PHLF. VWF-Ag facilitates the preoperative risk assessment for individuals with resectable HCC. Individuals who have vWF-Ag levels greater than 291% may be evaluated for other therapies, whereas those who have vWF-Ag levels of 182% or less are the most surgical candidates [36].

### 2.6. Fibrinogen and Fibrin

A glycoprotein called fibrinogen is present in vertebrate blood and may be coagulated by thrombin. Alpha, beta, and gamma polypeptide chains that make up human fibrinogen have their primary structures determined by amino acid and nucleic acid sequencing. As a bivalent entity, the entire molecule has a trinodular, dimeric structure. To reveal polymerization sites essential for the development of fibrin fibers and the ensuing clot formation, thrombin helps cleave short peptides off the amino termini of the alpha and beta chains. Fibrinogen’s main physiological function is to produce fibrin, which forms a hemostatic plug by binding platelets and certain plasma proteins [37].

In pathological conditions, the fibrin network can trap numerous erythrocytes and leukocytes, resulting in the formation of a thrombus that may block a blood vessel. Fibrinogen is essential for platelet aggregation and also interacts with various plasma proteins, although the full understanding of the biological implications of this interaction remains incomplete. Fibrin, on the other hand, serves as a crucial matrix for the regulation of fibrinolysis and aids in cell attachment during the process of wound healing [37].

In several cancers, the advancement of the tumor has been linked to elevated plasma fibrinogen levels. Another study attempts to define the therapeutic relevance of increased levels of plasma fibrinogen in HCC patients. According to the results, there was no significant difference seen between the plasma fibrinogen levels in patients with HCC and those in healthy controls or patients with cirrhosis [38]. High plasma fibrinogen levels were linked to tumor development, as seen by the advanced tumor stage, greater tumor size, increased tumor number, and vascular invasion, according to clinicopathological investigations. A worse prognosis and more advanced tumor progression are linked to elevated plasma fibrinogen levels. HCC patients may use plasma fibrinogen as a negative predictive biomarker [38].

It has been observed that a worse prognosis for many malignancies is linked to elevated plasma fibrinogen. Another study set out to determine if preoperative plasma fibrinogen in HCC patients was predictive of prognosis. A higher NLR, poorer MELD score, greater tumor diameter, vascular invasion, advanced Barcelona Clinic Liver Cancer stage, and poor–moderate pathological differentiation were all connected with elevated plasma fibrinogen. Furthermore, nomograms that took fibrinogen into account were created to forecast DFS and OS in patients with HCC [39].

In addition, subgroup studies revealed the predictive significance of fibrinogen in patients with HCC who had a single tumor and BCLC 0-A stage, as well as in patients with or without cirrhosis or high AFP levels. When a liver resection was performed on HCC patients, preoperatively increased plasma fibrinogen was found to be an independent predictor of poor prognosis [39].

According to reports, individuals with HCC have unknown and unpredictable survival rates, and hyperfibrinogenemia is a marker of poor prognosis in these patients. The current investigation sought to determine if plasma fibrinogen levels and overall survival in individuals with head cancer were related. To find the predicted risk variables for the rates of tumor recurrence and overall survival, univariate and multivariate analyses were carried out. Individuals in the high-fibrinogen-level group had a higher probability of advanced-stage HCC, portal vein invasion, and more tumors with bigger diameters and numbers than those in the low-fibrinogen-level group [40].

Patients in the high-fibrinogen group had a significantly worse long-term overall survival rate than patients in the normal-fibrinogen group. High plasma fibrinogen continued to be independently correlated with worse overall survival, according to the findings of the univariate and multivariate analyses. Furthermore, there was a significant correlation between elevated plasma fibrinogen levels and resistance to transarterial chemoembolization (TACE). As a result, elevated plasma fibrinogen may be a useful clinical biomarker for predicting prognosis in HCC patients. It was independently linked to an advanced HCC stage, a poor prognosis, and nonresponse to TACE [40].

Preoperative fibrinogen was shown to be associated with a greater hazard ratio and a higher overall survival in primary liver cancer (PLC) patients (>2.5 g/L). By activating the (protein kinase B) AKT/mTOR (mammalian target of rapamycin) pathway and promoting epithelial–mesenchymal transition (EMT), fibrinogen may simultaneously encourage hepatoma cell motility and invasion. Furthermore, mTOR inhibitors and phosphatase and tensin homolog (PTEN) overexpression may prevent the stimulation of fibrinogen on cell migration and invasion. The prognosis of individuals with primary liver cancer may be correlated with preoperative fibrinogen. In PLC patients, fibrinogen upregulation is progressively associated with an increase in mortality. By activating the PTEN/AKT/mTOR pathway, fibrinogen may induce EMT and facilitate hepatoma metastasis [41].

The development of HCC has been linked to an aberrant gene expression. Therefore, the plasma fibrinogen levels and fibrinogen gamma (FGG) mRNA expression status were researched in HCC patients. In the SMMC-7721 and HepG2 HCC cell lines, FGG was considerably upregulated at the mRNA level. When compared to nearby non-cancerous tissues as a reference, the FGG mRNA transcript was likewise upregulated in HCC tissues. It indicated considerably higher levels of plasma fibrinogen as compared to healthy individuals [42]. Furthermore, individuals with HCC showed a gradual increase in plasma fibrinogen as their tumor clinical stage increased. A positive plasma fibrinogen level was revealed to have a significant connection with the existence of tumor thrombosis by multivariate logistic regression analysis. Elevated plasma fibrinogen may be a helpful predictor of the clinical development of HCC patients, and FGG mRNA was expressed abnormally in HCC [42].

The choice of patients has a crucial role in enhancing the results of liver transplantation for hepatocellular cancer. Therefore, it is important to determine which biochemical markers may have an impact on a patient’s prognosis after liver transplantation. Preoperative fibrinogen and AFP (α-fetoprotein) levels are linked to recurrence, according to an analysis of the discovery cohort. After liver transplantation, preoperative fibrinogen and AFP can be used to predict the recurrence of HCC [43].

In patients with hepatocellular carcinoma undergoing liver transplantation, it examined the predictive significance of preoperative fibrinogen levels by developing a scoring model for tumor recurrence. Three independent risk variables for tumor recurrence were higher fibrinogen levels, macrovascular invasion, and more than three tumor nodules. In patients with HCC who have had liver transplants, an increased pretreatment plasma fibrinogen concentration is linked to tumor recurrence. With strong sensitivity and specificity, a novel scoring model predicted recurrence [44].

Patients with liver cirrhosis experience severe coagulopathy, bleeding, and clotting due to reduced coagulation factor production. Portal hypertension is induced by the coagulation system’s imbalance and thrombocytopenia. It is now known that chronic liver illness causes hemostasis to be rebalanced. Patients with cirrhosis have several coagulopathy diseases, and the liver plays a significant role in the synthesis of coagulation factors. Although fibrinogen levels are correlated with the degree of liver cirrhosis and decline with increasing disease severity, platelet and fibrinogen levels are unable to predict the extent of bleeding in these individuals [45].

A vital clotting factor, fibrinogen, is produced in the liver. Because of the widespread scarring and loss of functioning hepatocytes caused by liver cirrhosis, the liver may be less able to manufacture fibrinogen and other clotting components. This leads to a coagulopathy that is frequently observed in cirrhotic individuals and causes hypofibrinogenemia. Tumors may affect the liver’s remaining functions, which would further decrease the production of fibrinogen. Fibrinogen depletion results from gastrointestinal bleeding or other hemorrhagic consequences that are frequently caused by HCC. Fibrinogen levels were considerably lower in liver cirrhosis patients than in healthy controls, according to another work of research [46].

### 2.7. Tissue Factor

A significant correlation has been shown between tissue factor (TF) and cancer. A bad prognosis is linked to TF expression in cancer. The control of TF expression in cancer cells and the functions of TF and alternatively spliced (as) TF in tumor development and metastasis are outlined. Transcription factors, micro-ribonucleic acids, and a range of other signaling pathways control the expression of TF genes in cancer cells. By promoting the production of angiogenic factors including vascular endothelial growth factor and activating protease-activated receptor 2 signaling, the TF/factor VIIa complex promotes tumor development [47]. By improving integrin β1 signaling, AsTF promotes the development of tumors. Additionally, TF and asTF promote metastasis through a variety of thrombin-dependent and -independent pathways, one of which involves shielding tumor cells from natural killer cells. Tumor TF is the target of a revolutionary anticancer treatment that delivers cytotoxic medicines directly to the tumor. TF may be helpful in the identification, assessment, and management of cancer [47].

Tissue factor is a transmembrane glycoprotein that helps blood coagulate and is often overexpressed in several different types of cancer. TF expression is elevated in HCC and is linked with prognosis. It is still unknown, therefore, how TF functions and what chemical process drives the development of HCC. To find out how TF affected the proliferation of HCC cells, functional studies were conducted both in vitro and in vivo. To clarify the underlying processes, a panel of biochemical tests was employed [48].

By triggering the extracellular-signal-regulated kinase (ERK) and AKT signaling pathways, TF may encourage the formation of HCC both in vivo and in vitro. TF increased the epidermal growth factor receptor (EGFR) expression, while EGFR inhibition inhibited the development of HCC mediated by TF. Furthermore, in HCC tissues, TF protein expression and EGFR were linked. TF stimulates the formation of HCC by upregulating EGFR, and both TF and EGFR may be viable therapeutic targets for HCC [48].

Numerous studies show a strong correlation between the origin and progression of malignant tumors and TF, specifically tissue thromboplastin. It helps in blood coagulation, blood vessel creation, tumor metastasis and recurrence, and the control of cellular differentiation. The TF expression in HCC patients and its relationship to the disease’s clinical characteristics and prognosis was investigated. The levels of plasma TF in HCC were found to be substantially higher than in the controls. There was also a tight correlation seen between the levels of differentiation, tumor size, and the presence of hepatocirrhosis. In comparison to non-metastasis or non-tumor thrombus, there were significantly higher values in cases of lymphatic metastasis, extrahepatic metastasis, and portal tumor thrombus (PTT). However, there was no significant difference seen with various focus numbers or envelopes [49].

Compared to neighboring or normal tissues, HCC tissue had substantially higher positive rates and relative expression of TF mRNA. The relative expression intensity of the positive patients differed considerably depending on the tumor size, local invasion index, and metastasis index. Significantly greater than those in neighboring or normal tissue, the positive rates and relative expression intensities of TF protein were found in HCC tissue. The relative expression intensity revealed a significant difference in various tumor sizes, differentiation degree, and index of local invasion and metastasis in the patients with positive findings. The index of invasion and metastasis showed a strong correlation with the TF levels, which were considerably greater in the plasma and tissues of HCC patients. It was suggested that TF could be connected to HCC metastasis and differentiation [49].

Recent research indicates that TF might have a role in the angiogenesis and spread of tumors. It is uncertain how TF functions in HCC. In human HCC, another study assessed the potential correlations between TF expression and tumor invasiveness, vascular endothelial growth factor (VEGF) expression, microvessel density (MVD), and prognosis. Tumor MVD and the immunohistochemistry expression of TF in the tumors showed a strong correlation [50].

With a range of 67–2406 pg/mg total protein, the tumors’ median cytosolic TF protein level was 720 pg/mg. There was a noteworthy positive connection between the levels of TF and VEGF in the tumor cytoplasm. An advanced tumor stage, an unencapsulated tumor, microsatellite nodules, and venous invasion were all linked to high tumor cytosolic TF levels. Poor survival was independently predicted by a tumor cytosolic TF level greater than the median. It demonstrated a relationship between TF and HCC invasiveness and tumor angiogenesis. In patients with HCC, evaluating the expression of tumor TF may be helpful as a prognostic measure [50].

Cancer prognosis and progression are influenced by TF, the primary starter of the coagulation cascade. The TF–FVIIa interaction is reflected in the activated factor VII–antithrombin complex (FVIIa–AT), which is thought to be an indirect indicator of TF exposure. Elevated FVIIa–AT levels might be useful in identifying individuals whose disease involves a high TF expression and in predicting a greater risk of death in liver cancer [51].

The principal location of synthesis for almost all coagulation factors and numerous proteins involved in anticoagulation and fibrinolysis is the liver. A bleeding diathesis owing to a shortfall in the synthesis of coagulation factors and a procoagulant condition owing to abnormalities in the liver’s manufacture of anticoagulant factors are linked to both acute and chronic liver inflammation [52].

Between the group under study and other laboratory parameters, there was a statistically significant variation in tissue factor expression. Tissue factor was significantly positively correlated with creatinine, total bilirubin, AFP, aspartate aminotransferase (AST), alanine aminotransferase (ALT), international normalized ratio (INR), and activated partial thromboplastin time (APTT). Hemoglobin, albumin, and platelets showed a noteworthy negative connection with tissue factor. Pro-inflammatory cytokines are released when TF is applied, which intensifies the inflammatory process in the liver parenchyma. Thus, anti-inflammatory drugs may play a part in treating such individuals’ liver damage and may even be a therapeutic alternative [52].

The protein called tissue thromboplastin, or CD142, is in charge of starting the blood-clotting process. It does this, especially in reaction to vascular damage, by attaching itself to and activating factor VIIa, a plasma serine protease. Tissue factor plays an important role in hemostasis and is linked to pathological conditions related to hemostasis. It triggers the coagulation system in several thrombotic diseases, coagulopathies linked to sepsis, and other types of disseminated intravascular coagulation. Tissue factor has been shown in recent studies to have functions beyond hemostasis, such as influencing cell signaling, inflammation, vasculogenesis, tumor development, and metastasis [53].

While thrombosis is often linked to hepatocellular carcinoma, HCC is also linked to liver cirrhosis (LC), which results in hemostatic problems. Thus, utilizing a clot waveform analysis (CWA), hemostatic anomalies in patients with HCC were investigated. Using a CWA-activated partial thromboplastin time (APTT) and a small-amount-of-tissue-factor-induced FIX activation (sTF/FIXa) test, hemostatic anomalies were investigated in 88 samples from HCC patients, 48 samples from LC patients, and 153 samples from patients with chronic liver disorders (CH). HCC, LC, and CH did not differ substantially in their peak times on CWA-APTT, and HCC and CH had considerably greater peak heights on CWA-APTT than HVs and LC [54].

In HCC, the peak heights of the CWA-sTF/FIXa were notably higher. Stages B, C, and D had much longer CWA-APTT peak periods than stage A or response instances. On the receiver operating characteristic (ROC) curve, the fibrin formation height (FFH) of the CWA-sTF/FIXa and CWA-APTT demonstrated the best diagnostic capacity for LC and HCC, respectively. A study found that 13 HCC patients had thrombosis, and portal vein thrombosis and arterial thrombosis were often linked to HCC with LC and HCC without LC, respectively. The first derivative’s peak time × peak height on the CWA-sTF/FIXa had the best ROC diagnostic ability for thrombosis. The evaluability of HCC, especially its correlation with thrombotic problems and LC, can be improved by using the CWA-APTT and CWA-sTF/FIXa [54].

### 2.8. Tissue Plasminogen Activator

The plasminogen activator (PA) system is an extracellular proteolytic enzyme system that is associated with a number of physiological and disorders. Several pieces of evidence suggest that urokinase-type plasminogen activator (uPA), its receptor (uPAR), and plasminogen activator inhibitors-1 and -2 (PAI-1 and PAI-2) play a significant role in the growth and dissemination of malignancies among the various PA system components. When uPA and uPAR bind together, the extracellular matrix degrades, causing tumor cells to move from their main site of origin to a distant secondary organ [55]. This process also involves the activation of plasminogen to plasmin. The PA system’s components are excellent targets for diagnosis, prognosis, and treatment for lower cancer-related morbidity and mortality since they exhibit changed expression patterns in several prevalent malignancies [55].

Urokinase plasminogen activator (uPA) is classified as a serine protease that is instrumental in facilitating the transformation of plasminogen into its active derivative, plasmin, thereby contributing significantly to the fibrinolysis mechanism. Although uPA is not categorized as a coagulation factor, it constitutes an integral component of the fibrinolytic system; nevertheless, this process, referred to as fibrinolysis, is crucial for maintaining equilibrium within the coagulation system. uPA is known to contribute to tissue invasion together with matrix metalloproteinases (MMPs), especially in cancer metastasis. uPA, a protease that breaks down the extracellular matrix (ECM), is a major contributor to HCC invasion and metastasis. The clinical importance of uPA in HCC was confirmed by looking at its expression in the disease using The Cancer Genome Atlas (TCGA) database. The tumor size, differentiation grade, and absence of tumor encapsulation were all significantly associated with the overexpression of uPA in HCC [56]. For patients with high levels of uPA expression, the prognosis was poor. Lastly, it discovered that uPA was overexpressed in HCC and linked to characteristics of malignant HCC, including tumor size, differentiation grade, and absence of tumor encapsulation. Patients with a higher uPA expression had lower survival times. It has the potential to be an HCC predictive biomarker [56].

HCC is the liver’s most common primary malignant tumor. The accumulation of chromosomal changes provides the critical stages of carcinogenesis, which have been clarified by molecular genetic studies. During invasion and metastasis, uPA is generated as a crucial enzymatic process that degrades the extracellular matrix. The kinetics of uPA cellular expression during this process were investigated by immuno-histochemistry on liver slices from a rat HCC using specific polyclonal antibodies against uPA [57].

The onset of many hyperplastic nodules in the early phases of the model-induced neoplastic transformation was followed by the lesions eventually developing into well-differentiated HCC. A gradual increase in uPA expression, which peaked five and six months after the carcinogenic medicines were administered, was linked to the morphological alterations of premalignant and malignant lesions. Among the examined enzymatic markers, gamma-glutamyl transpeptidase showed a link with the histology results. Not only should uPA expression be considered an indication of metastasis, but it may also be linked to the development of biliary ducts and arteries as well as the early phases of the neoplastic transformation [57].

Plasma u-PA levels were statistically higher in the group of patients with decompensated liver cirrhosis than in the group with compensated liver cirrhosis with HCC, decompensated liver cirrhosis with HCC, and compensated liver cirrhosis. In all three of these latter patient groups, the u-PA levels were moderately and substantially elevated, but not statistically different from those in the chronic hepatitis group and the healthy volunteers. The kind, quantity, and size of HCC tumors invaded, as well as the u-PA plasma level, did not correlate [58].

In three groups without an associated HCC, significant correlations were found between the results of seven different liver function tests and u-PA plasma levels; correlations were found between u-PA antigen and serum total bilirubin and serum total bilirubin, and between u-PA antigen and prothrombin time (%), hepaplastin test (%), serum cholinesterase, serum albumin, and serum total cholesterol, negatively. These findings imply that, in contrast to HCC invasion, plasma u-PA is linked to a decline in liver function [58].

The extracellular-matrix-degrading protease uPA contributes to the invasion and spread of cancer. While there is strong evidence that the uPA expression in various solid tumors is a clinically significant biomarker, its significance in HCC remains unclear. Before surgery, it assessed the predictive significance of serum uPA in patients with HCC undergoing curative resection. OS was considerably shorter in patients with greater pretreatment blood uPA. Elevated uPA levels were substantially more common in patients with thrombocytopenia, hypoalbuminemia, and liver cirrhosis [59].

The results of a multivariate Cox regression analysis showed that pathology stage III/IV, vascular invasion, and high pretreatment serum uPA were independent predictive variables for OS. Serum AFP and uPA together demonstrated a greater ability to predict OS in additional stratified studies. A greater expression of uPA is linked to a greater death rate, making it a clinically important biomarker for HCC patients undergoing curative resection. This further emphasizes the potential use of uPA as a therapeutic target to facilitate more effective treatment plans [59].

### 2.9. Soluble Urokinase-Type Plasminogen Activator Receptor

The expression of uPAR is low in healthy tissues and high in malignant tumors, making it a promising target for cancer treatment. Malignant tumor invasion and metastasis are intimately associated with uPAR. The disintegration of the extracellular matrix (ECM), tumor angiogenesis, cell proliferation, and apoptosis are all significantly impacted. Additionally, uPAR is associated with tumor cells’ multidrug resistance (MDR), which is a critical determinant of tumor aggressiveness and prognosis. To inhibit tumor development, metastasis, and treatment resistance, several uPAR-targeted antitumor therapeutic drugs have been created [60].

uPAR, a key protein in the plasminogen activation pathway, is well-established to be essential for the invasion and spread of many cancer cells. Urokinase plasminogen activator and its receptor have been shown to be overexpressed in a wide range of tumor cells. Expression of uPA/uPAR is necessary for tumor cell invasion and metastasis in breast cancer. It is still unknown, nevertheless, what mechanism causes uPA/uPAR expression in cancer cells [61].

uPAR signals were detected and these signals were mostly located in the cytoplasm of the tumor cells and tended to be in front of invasive foci. Hepatocytes close to the portal tracts showed modest uPAR mRNA and protein signals in non-neoplastic instances, such as chronic active hepatitis and cirrhosis, indicating that this protein may be involved in these conditions. uPAR expression may be a potential predictor of these parameters, and uPAR is involved in the invasion and metastasis of HCC, at least in the early stages [61].

Patients with intermediate-stage HCC or liver metastases are often treated with transarterial chemoembolization (TACE). It is still difficult to determine who would be the best candidates for TACE treatment. There are currently no data on the role of the suPAR in TACE, despite its recent development as a prognostic marker in cancer patients [62].

When comparing liver cancer patients to healthy controls, serum levels of suPAR were demonstrably higher in the former group. Circulating suPAR levels were similar in patients who did not exhibit an objective tumor response to TACE treatment. suPAR serum concentrations did not rely on tumor type, leukocyte count, or C-reactive protein. However, they did correlate with creatinine serum concentrations. Baseline suPAR serum levels offer crucial details about the postinterventional results of TACE-treated liver cancer patients [62].

### 2.10. Plasminogen Activator Inhibitor-1

Even though PAI-1 is a key inhibitor of urokinase (uPA), a vast clinical database study has confirmed that it is strongly expressed in various tumor biopsy tissues or plasma when compared to controls. Of greater significance, PAI-1, either alone or in conjunction with uPA, has been found to be a predictor of disease progression and recurrence in some cancer types [63].

PAI-1 has been shown to increase tumor vascularization, which, in turn, promotes cell dispersion and tumor metastasis, in addition to its significant functions in cell adhesion, migration, and invasion. Furthermore, a variety of tumor-promoting variables influence how PAI-1 is expressed and activated, which enhances PAI-1’s pro-tumorigenic properties. PAI-1 is a potential target for therapeutic intervention in particular cancer treatments. A few PAI-1 inhibitors are presently being studied for use in cancer treatment; these might eventually lead to the development of novel anticancer drugs [63].

It has been revealed that the uPA system’s plasminogen activator inhibitor-1 plays a critical role in the initiation of several cancer types. Eight introns and nine exons make up the PAI-1 gene, which is found on chromosome 7. The most prevalent variant (4G/5G) in this highly polymorphic gene influences PAI-1 production and, in turn, the amount of the protein that is circulating. In Egyptian patients with HCC and chronic HCV, the genotype distribution and allelic frequency of the PAI-1 4G/5G polymorphism were investigated [64].

It was also assessed how the PAI-1 4G/5G polymorphism affected the levels of PAI-1 in serum. There was no statistically significant difference between the two research groups in the frequency of alleles (5G and 4G) or the genotypic distributions of the 4G/5G polymorphism (5G/5G, 4G/4G, 4G/5G, and 4G/4G + 4G/5G) (*p* > 0.05). Additionally, for any of the several genotypes of the 5G/4G polymorphism, there was no statistically significant difference in blood levels of PAI-1 between patients with HCC and patients with HCV (*p* > 0.05), nor across genotypes within the same group. Patients with persistent HCV infection in Egypt may not have the PAI-1 4G/5G polymorphism as one of the underlying genetic reasons for hepatocarcinogenesis [64].

Although its role in promoting or impeding tumor growth has been debated, PAI-1 is a serine protease inhibitor that inactivates uPA and controls the extracellular matrix’s breakdown. A vector capable of expressing an antisense PAI-1 transcript was used to transfect HLE cells to assess the role of PAI-1 in HCC cell invasion and proliferation. Seven stably transfected clones (PAI-1^−^) were examined, and the results showed an enzyme-linked immunosorbent assay of 63% in the cellular PAI-1 antigen level and an 81% drop in PAI-1 mRNA by a Northern blot analysis [65].

There was no change in the amounts of released PAI-1 and PAI-2. Cellular uPA activity increased by 54% without changing the protein level or the ELISA detection of secreted uPA activity. Smaller cytoplasmic processes and a spindle shape were the results of PAI-1 anti-sense in HLE cells. When PAI-1 was enforced, the invasion and growth rates in PAI-1^−^ cells increased by 75% and 82%, respectively. uPAR is crucial for HCC invasion and metastasis, at least in the early stages, and its expression may be a potential predictor of these variables. uPAR is crucial for HCC invasion and metastasis, at least in the early stages and its expression may be a potential predictor of these outcomes [65].

When treating cancer, targeting the tumor stroma is a crucial tactic. Tumor-associated macrophages (TAMs) and cancer-associated fibroblasts (CAFs) are two key elements of the tumor microenvironment (TME) in HCC that might accelerate tumor growth. The upregulation of plasminogen activator inhibitor 1 (PAI-1) in HCC is indicative of unfavorable tumor behavior and prognosis. The interactions among cancer cells, TAMs, and CAFs, as well as the roles of PAI-1 in HCC, have not yet been thoroughly examined [66].

Key paracrine factors and macrophage polarization were evaluated in the current study when the cells interacted with cancer cells and CAFs. Through upregulating the mRNA expression levels of CD163 and CD206 and downregulating the mRNA expression and secretion of IL-6 by macrophages, it was found that cancer cells and CAFs induced the M2 polarization of TAMs. TAMs generated from cancer cells and CAFs helped HCC cells invade and proliferate [66]. The expression of PAI-1 was also increased in TAMs after stimulation with a CAF-conditioned media, and it promoted the malignant phenotype of HCC cells via controlling the epithelial–mesenchymal transition. CAFs were the primary makers of C X C pattern chemokine ligand 12 (CXCL12) in the TME, and CXCL12 aided in inducing TAMs to secrete PAI-1. It showed that, through CXCL12, CAFs induced the synthesis of PAI-1 and promoted macrophage M2 polarization. Moreover, it was discovered that the TAMs’ production of PAI-1 accelerated the HCC cells’ malignant tendencies. Consequently, these variables could be targeted to prevent tumor cells, CAFs, and TAMs from communicating with one another [66].

The biological roles of PAI-1 in HCC are still up for debate. It also needs more research since its expression, clinicopathologic importance, and predictive value in HCC have not been well-studied. It was discovered that, compared to nearby liver tissues, HCC had noticeably greater levels of PAI-1 expression. Furthermore, individuals with numerous lesions had a higher frequency of elevated PAI-1 expression. The results of the univariate analysis indicated a negative correlation between PAI-1 expression, and overall and relapse-free survival [67].

While the PAI-1 expression in the multivariate Cox regression test did not show statistical significance, when combined with the TNM stage, it was able to discriminate between survival and recurrence and functioned as an independent prognostic factor. It was also discovered that SERPINE1, the gene encoding PAI-1, was predictive in the online datasets of HCC and liver cancer utilized. According to the scientific literature findings, a long-term poor prognosis and adverse biological behavior in HCC were predicted by increased PAI-1 expression [67].

### 2.11. Antithrombin

Antithrombin (AT) is a glycoprotein classified within the family of serine protease inhibitors (serpins) and constitutes an integral component of the anticoagulation mechanism. It serves to inhibit coagulation factors, including thrombin and Factor Xa, thereby obstructing excessive thrombus formation and preserving hemostatic equilibrium within the vascular system. This characteristic is of paramount significance in the prophylaxis and management of thrombotic disorders. Antithrombin-III (AT-III) is another name for antithrombin. It is a liver-produced protein with 464 amino acids. There are three disulfide linkages and four potential glycosylation sites in all. A key factor in the development of HCC is inflammation. Through its anti-inflammatory properties, it investigated if antithrombin contributes to slowing the progression of HCC. AT controls neutrophil/IL-8 signaling to slow the growth of HCC [68].

During liver failure, ischemia-reperfusion damage, vascular endothelial dysfunction, and disseminated intravascular coagulation (DIC), the serum activity of AT-III which is generated in the hepatocytes declines. The blood AT-III levels on the first postoperative day may be a prognostic factor for patients with HCC [69].

It is uncertain if serum AT-III has any predictive significance in HCC, although several studies have demonstrated its anti-inflammatory properties. It examined the impact of preoperative AT-III levels on the prognosis of patients who had HCC and underwent hepatectomy. There is a correlation between preoperative antithrombin III levels and the prognosis of individuals with HCC. AT-III might help forecast how patients with HCC would do following a curative hepatectomy [70]. It is useful to determine the pathological background and forecast postoperative liver failure or dysfunction based on the serum AT-III level before hepatectomy. It should be feasible to employ AT-III as an extra liver function indicator [71].

Antithrombin-III levels in plasma are influenced by liver function. Patients with portal vein thrombosis (PVT) and low plasma AT-III levels benefit from AT-III. The prognosis of individuals with cirrhosis-associated PVT is uncertain, nevertheless, concerning these values. Patients who died from liver failure had a substantially lower plasma AT-III level than those who died from HCC, although there was no significant difference in the Child–Pugh or albumin–bilirubin (ALBI) scores between the two groups. A poor survival result was independently predicted by low plasma AT-III levels. Death, especially death from liver failure, may be linked to low plasma AT-III levels regardless of liver function [72].

Cirrhosis patients may be more likely to hemorrhage because of thrombocytopenia, platelet dysfunction, reduced hepatic production of procoagulant proteins, and increased portal pressure. Patients with liver illness may experience abnormal bleeding due to thrombocytopenia, the decreased hepatic production of coagulation proteins, the higher portal pressure and varix development, or qualitative platelet dysfunction. The two main causes of thrombocytopenia in liver illness are decreased platelet production as a result of decreased thrombopoietin production and splenic sequestration [73].

Certain infections and alcohol can inhibit the marrow. In individuals with liver illness, especially those with autoimmune liver disease or chronic hepatitis C, immune thrombocytopenia (ITP) may happen concurrently. Antibiotics, immunosuppressants, and interferon are among the medications used to treat liver illness or its consequences that might result in thrombocytopenia. The periprocedural therapy of thrombocytopenia in liver disease is contingent upon the bleeding risk of the surgery as well as the unique features of each patient [73].

It is unusual for patients who need low-risk operations or have a platelet count more than or equal to 50,000/µL to need platelet-directed treatment. If a patient needs a high-risk surgery and has a platelet count below 50,000/µL, platelet-directed treatment should be explored, particularly if the patient has other bleeding risk factors, such as irregular bleeding with previous hemostatic problems [73].

Platelet sequestration in the spleen and reduced hepatic thrombopoietin production are the main causes of thrombocytopenia in liver cirrhosis. For cirrhotic individuals with severe thrombocytopenia, several therapy options are now available, such as platelet transfusion, interventional partial splenic embolization, and surgical splenectomy. Although thrombopoietin agonists and targeted medicines are non-invasive alternatives for treating cirrhosis-related thrombocytopenia, clinical trials are being conducted to see if they may improve thrombocytopenia in cirrhotic patients [74].

Treatment strategies that safely and efficiently increase platelet counts may have a big impact on how these individuals are treated. The oral platelet growth factor eltrombopag is one of several potential new drugs being developed for the prevention and/or treatment of thrombocytopenia. These drugs activate thrombopoietin (TPO) and raise platelet counts. Enhancing platelet counts might greatly decrease the requirement for platelet transfusions and make it easier for patients with liver disease to receive interferon-based antiviral medication and other medically prescribed therapies [75].

Complex changes in the hemostatic system are common in patients with cirrhosis. Although the platelet count, prothrombin time, and fibrinogen level are standard diagnostic tests for hemostasis in cirrhosis and indicate a propensity for bleeding, it is now generally acknowledged that these measures do not accurately represent hemostatic competence in this group. Instead, a rebalanced hemostatic system with hypercoagulable components seems to be present in cirrhosis patients [76].

According to current clinical guideline papers, the routine correction of hemostasis test results by the use of fresh frozen plasma or platelet concentrates, for example, is not recommended to prevent spontaneous or procedure-related bleeding [76]. There are, however, few guidelines on how to treat cirrhosis patients who are bleeding actively. Three typical bleeding situations are variceal bleeding, post-procedural bleeding, and bleeding in a critically ill cirrhosis patient. Even in the presence of noticeably abnormal platelet counts and/or prothrombin times, prohemostatic therapy is not the first line of treatment for bleeding patients with cirrhosis because these patients typically have adequate hemostatic competence and because bleeding complications may not be related to hemostatic failure. In patients with cirrhosis who are bleeding, the literature results justify the use of a limited approach to prohemostatic medication [76].

Changes in purinergic signaling pathways, a decreased synthesis of vitamin K-dependent and -independent clotting factors and anticoagulant factors, increased platelet destruction due to hypersplenism, and decreased platelet production are other hypothesized mechanisms causing this hemostatic imbalance [77]. The pathophysiology of impaired hemostasis and thrombophilia in cirrhosis is updated. The common thrombotic consequences of cirrhosis, portal vein thrombosis (PVT), and venous thromboembolism (VTE), which cause significant morbidity and death, are discussed. New preventative measures and existing therapeutic alternatives to control the increased risk of thrombosis in cirrhosis while preventing hemorrhagic consequences are investigated [77].

A rare but serious and potentially fatal condition among patients of solid organ transplants is thrombotic microangiopathy (TMA). However, there have not been many documented studies on TMA in recipients of living-donor liver transplants (LDLTs). The reduced administration of fresh frozen plasma and sensitization to HLA are strongly associated with TMA, according to a multivariate analysis (odds ratio [OR]: 2.6 and 16.1, respectively) [78]. A comparison of the clinical variables revealed that a poor response to treatment, delayed diagnosis and/or treatment, and late start (>30 days) are all linked to poor outcomes. The prognosis may be improved by early diagnosis and the start of intensive therapy because it is challenging to avoid TMA in LDLT patients [78].

Primary platelet (PLT) aggregation and endothelial damage are enhanced in thrombotic microangiopathy, a microvascular occlusive disease. In a study, seven recipients of living-donor liver transplants (LDLTs) out of 206 patients who were assessed developed the TMA-like condition (TMALD). A histopathological examination of liver samples following LDLT showed clear variations based on the results [79].

All of the patients’ qualitative analyses of antibodies against metalloproteinase with thrombospondin type 1 motif and a disintegrin-like domain (ADAMTS-13) came out negative. Red cell fragmentation, microhemorrhagic macules, and platelet counts were early indicators of TMALD suspicion following LDLT. In every instance, the vWF/ADAMTS-13 ratio demonstrated a distinct diagnostic value, even though the absolute values of vWF and ADAMTS-13 did not always correspond to TMALD [79].

While thrombotic microangiopathy is acknowledged as a poor prognosis risk following liver transplantation, it is unknown how exactly TMA would affect outcomes. Living-donor liver transplants were used in all instances, and the patient survival rates were 66.9%, 64.6%, and 62.2% one, three, and five years following transplantation, respectively. Only the necessity of renal replacement therapy during TMA treatment was shown to be substantially associated with a poor outcome following the onset of TMA in a multivariate analysis. Overall, TMA’s results were not very good. Perhaps the sole factor contributing to the poor prognosis following the formation of TMA is the advancement of renal impairment despite extensive therapy [80].

Liver cirrhosis and HCC are closely related, as cirrhosis is a major risk factor for the development of HCC. The cirrhotic liver creates a microenvironment rich in oxidative stress, cytokines, and growth factors, all of which promote carcinogenesis. Coagulation factors play a significant role in the pathogenesis of HCC through their involvement in inflammation, angiogenesis, and tumor progression. In chronic liver disease, the liver’s capacity to produce coagulation factors is impaired, leading to a disrupted hemostatic balance. This creates a prothrombotic or hemorrhagic environment, both of which can contribute to liver damage and cancer development. Targeting the coagulation cascade may offer novel therapeutic strategies to prevent or treat HCC.

## 3. Conclusions

**Figure 1:**
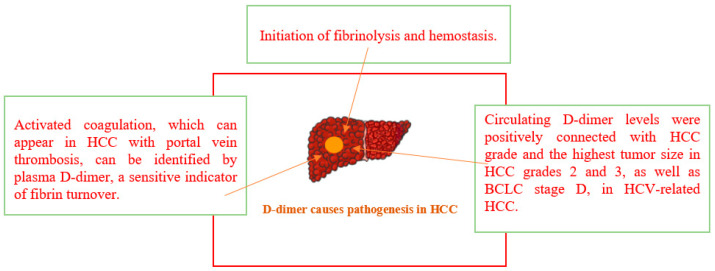
Pathogenesis of d-dimer in HCC.

**Figure 2:**
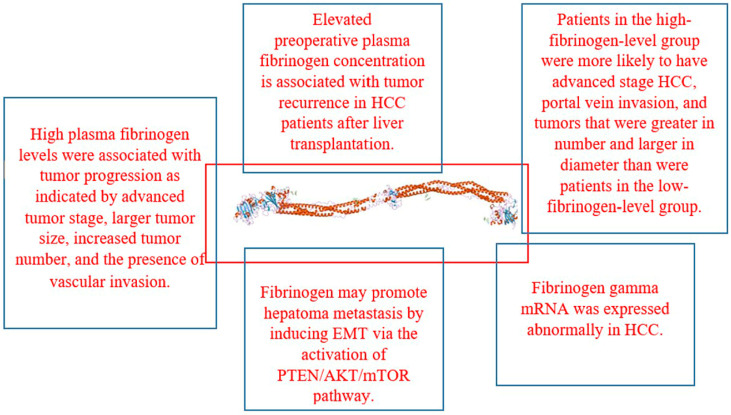
Pathogenesis of fibrinogen in HCC.

**Figure 3:**
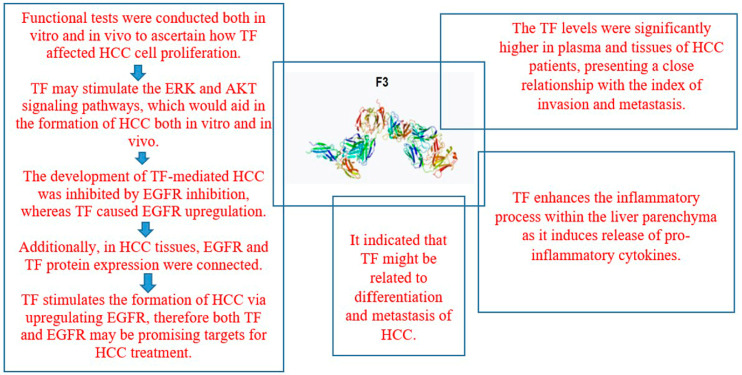
Pathogenesis of tissue factor in HCC.

**Figure 4:**
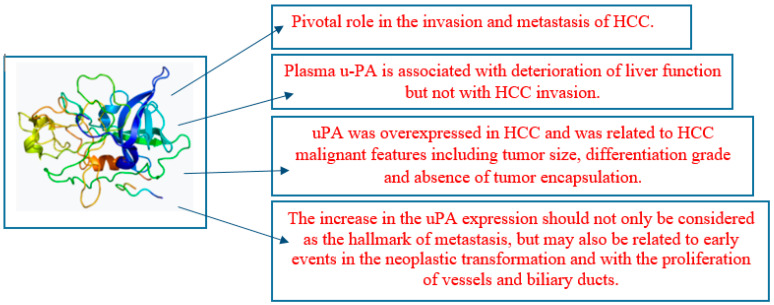
uPA causes pathophysiology in HCC.

This review concludes that members of the coagulation system such as P-selectin, thrombomodulin, d-dimer, prothrombin, von Willebrand factor, fibrinogen and fibrin, tissue factor, tissue plasminogen activator, plasminogen activator inhibitor-1, soluble urokinase-type plasminogen activator receptor, and antithrombin play significant role in the pathophysiology of HCC as explained in Figure 1, Figure 2, Figure 3 and Figure 4 and Table 1 and Table 2. Managing coagulation abnormalities in HCC involves a multifaceted approach that addresses both the underlying liver disease and the specific coagulation issues. The regular monitoring of coagulation parameters is important in patients with HCC, especially in those undergoing treatment or surgical procedures.

## Figures and Tables

**Table 1:** Circulating levels of coagulation factors in the pathogenesis of HCC.

Coagulation Factors	Circulating Levels in HCC
Urokinase-type plasminogen activator (fibrinolytic system)	↑
Tissue factor levels	↑
Antithrombin (anticoagulation system)	↓
Plasminogen activator inhibitor-1	↑
Thrombomodulin	↑
Fibrinogen levels	↑
von Willebrand factor	↑
Des-gamma-carboxy-prothrombin	↑
P-selectin	↑
d-dimer	↑

(↑) increased levels, (↓) decreased levels.

**Table 2:** Pathophysiological aspects of coagulation factors in HCC.

Coagulation Factors	Pathophysiological Aspect in HCC
Thrombomodulin	In HCC, TM could influence the spread of malignancy. Zinc finger E-box binding homeobox-1 (ZEB1) levels may increase and E-cadherin levels may be decreased if TM expression is downregulated.
Des-γ-carboxyl prothrombin	DCP functions for HCC cell lines as an autologous mitogen. DCP-induced cell proliferation may be mediated by the Met–Janus kinase 1–STAT3 (signal transducer and activator of transcription 3) signaling pathway. When combined with alpha-fetoprotein, prothrombin produced by vitamin K absence or antagonist-II (PIVKA-II) can be utilized as a tumor marker for the early diagnosis of HCC in individuals with persistent HBV infection.
von Willebrand factor	In addition to being linked to hepatic spare capacity and HCC, VWF has also been linked to angiogenesis and apoptosis. VWF: Ag may help predict the development of HCC and is linked to liver fibrosis. VWF is a potentially helpful biomarker to identify severe liver fibrosis and forecast the emergence of HCC. vWF-Ag levels and tumor size in the removed specimens were significantly correlated.
Urokinase-type plasminogen activator receptor	Crucial in the early stages of HCC invasion and metastasis.
Plasminogen activator inhibitor-1	PAI-1 inhibits invasion and proliferation; therefore, evaluating the invasiveness of HCC cells requires a balance between uPA and PAI-1 expression. It was proposed that cancer-associated fibroblasts stimulated macrophage M2 polarization and triggered the release of PAI-1 through C-X-C motif chemokine ligand 12. The tumor-associated macrophage’s production of PAI-1 increased the HCC cells’ malignant behavior. As a result, these elements may be the focus of blocking the communication between tumor cells, cancer-associated fibroblasts, and tumor-associated macrophages.

## Data Availability

All data generated or analyzed during this study are included in this article.
